# Differences in antimicrobial resistance gene abundance and microbial diversity of the gut microbiome in patients on antibiotics enrolled in a clinical trial

**DOI:** 10.1177/20499361251337597

**Published:** 2025-06-05

**Authors:** Adam G. Stewart, Patrick N. A. Harris, Rikki M. A. Graham, Amy V. Jennison, Sanmarie Schlebusch, Asha Kakkanat, Tiffany Harris-Brown, David L. Paterson, Brian M. Forde

**Affiliations:** Centre for Clinical Research, Faculty of Medicine, The University of Queensland, Royal Brisbane and Women’s Hospital Campus, Brisbane, QLD, Australia; Central Microbiology, Pathology Queensland, Royal Brisbane and Women’s Hospital, Brisbane, QLD, Australia; Centre for Clinical Research, Faculty of Medicine, The University of Queensland, Royal Brisbane and Women’s Hospital Campus, Brisbane, QLD, Australia; Central Microbiology, Pathology Queensland, Royal Brisbane and Women’s Hospital, Brisbane, QLD, Australia; Q-PHIRE Genomics and Public Health Microbiology, Queensland Health Forensic and Scientific Services, Coopers Plains, Brisbane, QLD, Australia; Q-PHIRE Genomics and Public Health Microbiology, Queensland Health Forensic and Scientific Services, Coopers Plains, Brisbane, QLD, Australia; Centre for Clinical Research, Faculty of Medicine, The University of Queensland, Royal Brisbane and Women’s Hospital Campus, Brisbane, QLD, Australia; Central Microbiology, Pathology Queensland, Royal Brisbane and Women’s Hospital, Brisbane, QLD, Australia; Q-PHIRE Genomics and Public Health Microbiology, Queensland Health Forensic and Scientific Services, Coopers Plains, Brisbane, QLD, Australia; Q-PHIRE Genomics and Public Health Microbiology, Queensland Health Forensic and Scientific Services, Coopers Plains, Brisbane, QLD, Australia; Centre for Clinical Research, Faculty of Medicine, The University of Queensland, Royal Brisbane and Women’s Hospital Campus, Brisbane, QLD, Australia; Centre for Clinical Research, Faculty of Medicine, The University of Queensland, Royal Brisbane and Women’s Hospital Campus, Brisbane, QLD, Australia; ADVANCE-ID, Saw Swee Hock School of Public Health, National University of Singapore, Singapore, Singapore; Centre for Clinical Research, Faculty of Medicine, The University of Queensland, Royal Brisbane and Women’s Hospital Campus, Brisbane, QLD, Australia

**Keywords:** antibiotic resistance, antibiotics, clinical trial, gut microbiome

## Abstract

**Background:**

Understanding how the gut microbiome adapts on exposure to individual antibiotics, with respect to antimicrobial resistance gene (ARG) enrichment, is important.

**Objectives:**

To characterise the changes that occur in the gut microbiome of patients enrolled in an antibiotic clinical trial and to propose methods in which to embed gut microbiome analysis into clinical trials.

**Design:**

This was a prospective cohort study of hospitalised patients who were successfully enrolled and randomised into two clinical trials between January 2021 to December 2021.

**Methods:**

Adult patients admitted to the hospital with a bloodstream infection have been randomised to receive either benzylpenicillin, ampicillin, cefazolin, ceftriaxone, piperacillin-tazobactam or meropenem at a single institution. Faecal specimens were collected at enrolment and every second day until discharge. Each specimen underwent DNA sequencing to determine microbial diversity and ARG abundance.

**Results:**

Ten patients (including six females) were included. DNA concentration and sampling quality were markedly lower for rectal swabs compared to stool samples. Relative abundance of *Enterococcus faecium* was increased in individual patients where treatment included ampicillin, meropenem and piperacillin-tazobactam. Piperacillin-tazobactam also increased the abundance of key beta-lactamase genes (*bla*_SHV-100_, *bla*_OXA-392_, *bla*_CMY-18_). Ampicillin increased the abundance of *bla*_TEM-1A_. There were no extended-spectrum beta-lactamase (ESBL) or carbapenemase genes detected in our study. The presence of key anaerobes such as *Clostridium* and *Bifidobacterium* species appeared to play an important role in colonisation resistance of *E. faecium* and *Clostridioides difficile*.

**Conclusion:**

Differential changes in anaerobic bacterial genera on exposure to antibiotics may be a key determinant of colonisation resistance. The pre-analytical phase of microbiome analysis is a critical factor in data quality and interpretation.

## Introduction

The global burden of antimicrobial resistance continues to rise and is largely driven by antimicrobial use.^1^ There exists a dire need for new management strategies to mitigate this growing risk to patient and public health outcomes. Despite being crucial for the treatment of severe infections, antimicrobial use leads to unintended consequences on the human microbiome.^2^ Indeed, inappropriate and excessive use of antimicrobials also contributes to antimicrobial resistance. Within the gut microbiome, administration of broad-spectrum antimicrobials can cause a rapid and significant drop in taxonomic richness, diversity and evenness.^2,3^ Moreover, these compositional changes can remain long after cessation of antimicrobials.^4^ In addition to changes in diversity, an increase in the abundance of antibiotic-resistance genes belonging to the resistome is observed.^5^ This includes problematic antimicrobial resistance determinants such as those encoding extended-spectrum beta-lactamases (ESBL) and carbapenemases in Gram-negative bacteria, as well as vancomycin resistance genes (vanA and vanB) in Enterococcus spp. Colonisation resistance is the reverse process whereby the microbiota inhibits the expansion of Enterobacterales and enterococci in the gut lumen and is promoted by taxonomic diversity (including the presence of anaerobic bacteria).^6^

It remains unclear what effect each antimicrobial has on the gut microbiome of individual patients with respect to bacterial community profiling, taxonomic diversity and antimicrobial resistance gene (ARG) abundance. Difficulties exist in carrying out such *in vivo* studies with no agreed-upon methodology (including statistical analysis) for the assessment and comparison of clinical specimens undergoing metagenomic sequencing.^7^ Significant differences in richness and diversity have been observed within and between healthy patient stool specimens. Gaining a more refined understanding of the effect of the individual antibiotic over time, on the type and degree of change in the gut microbiome, will better inform decision making on antimicrobial prescribing. Moreover, developing an agreed-upon framework in which to embed gut microbiome analysis into contemporary clinical trials will help to compare clinically relevant antibiotic-related adverse events.^8^ Here, we aim to characterise the changes that occur in the gut microbiome of patients enrolled in an interventional clinical trial receiving a single antibiotic, in addition to proposing a methods structure in which to embed gut microbiome analysis into clinical trials.

## Methods

### Clinical data

Adult patients admitted to the hospital with bloodstream infection at the Royal Brisbane & Women’s Hospital who had been enrolled and randomised into an interventional antibiotic clinical trial were identified prospectively for inclusion in the study between January 2021 to December 2021. Two such trials were enrolled during the study period, GAMECHANGER (ClinicalTrials.gov NCT03869437) and BALANCE (Clinical Trials.gov NCT03005145). All patients enrolled in either of these trials at our centre were eligible for inclusion in our study. Stool samples or rectal swabs were collected at baseline and every 2–3 days thereafter until discharge from the hospital. Collection of stool samples was given preference over rectal swabs by the study team. Patient demographic, microbiological and clinical data were collected prospectively. This study was approved by the Royal Brisbane & Women’s Hospital Human Research Ethics Committee (LNR/2020/QRBW/65029). *Written informed consent for inclusion in our study was obtained from participants*.

### Sample handling and storage

Fresh faeces specimens were collected in a non-sterile container, and rectal swabs were collected in a commercial system (MWE DRYSWAB™). Faeces specimens were visually inspected, and their appearance was recorded (e.g., watery). Rectal swabs were visually inspected for discolouration immediately post-sampling (re-collection was performed if no discolouration was evident). Samples were stored at 4°C for <72 h before being transported to our reference laboratory and ultimately stored at −80°C prior to DNA extraction.

### Sequencing methods

DNA extraction was performed using the QIAamp Power Fecal Pro DNA kit (Qiagen, Clayton, Victoria). Briefly, each sample was mixed with lysis buffer and sterile zirconia beads as per the manufacturer’s instructions. After bead-beating, using a modified Vortex Genie vortex mixer for 10 min (faeces) or 30 min (swabs) at room temperature, the samples were centrifuged for 5 min at 13,200 rpm and the supernatant was further treated as per the manufacturer’s recommendations. A blank control was included with each extraction run and subsequently sequenced for quality control and monitoring of possible contamination. Library preparation and sequencing were performed as previously described by Schlebusch et al.^9^

### Bioinformatic analysis

Human reads were quantified and removed by mapping trimmed reads to the human genome using bowtie2.^10^ The raw reads were trimmed which were then used as input to MGmapper.^11^ An acquired AMR gene database (ResFinder version 8 (1)) was used to annotate properly paired reads (MGmapper option fullmode) where each read pair had sequence coverage of at least 80% compared with the length of the trimmed reads. This was the only filter that was applied to discard a read pair. An AMR gene database (ResFinder) was used to annotate properly paired reads.^12^ To enable multiple database hits, AMR genes were mapped using the fullmode option in MGmapper for the most optimal abundance calculation. Genomic annotation was performed by identifying the best hit (MGmaper option bestmode) for a pair of reads when aligned against a range of reference sequence databases. The total bacterial read count for a sample was calculated as the sum of read counts from each of the bacteria-related databases. The total fraction of unmapped reads for all samples was used to translate the percentage of unmapped reads into Z-scores (i.e., the number of standard deviations from the mean). Community profiling was performed using Kraken.^13^ Relative abundance of AMR genes and bacterial genera was calculated as fragments per kilobase of transcript per million mapped reads (FPKM). This was done to account for both sample-wise sequencing depth differences and size-dependent probability of observing a reference. For AMR genes, FPKM was calculated on each individual ResFinder reference sequence. FPKMs were subsequently summed up across categories to bacteria genera, drug class level and highly homologous AMR gene groups. Alpha and beta diversity and richness were calculated on rarefied versions of the resistance and bacterial count matrices.

## Results

### Clinical data

**Figure 1.**
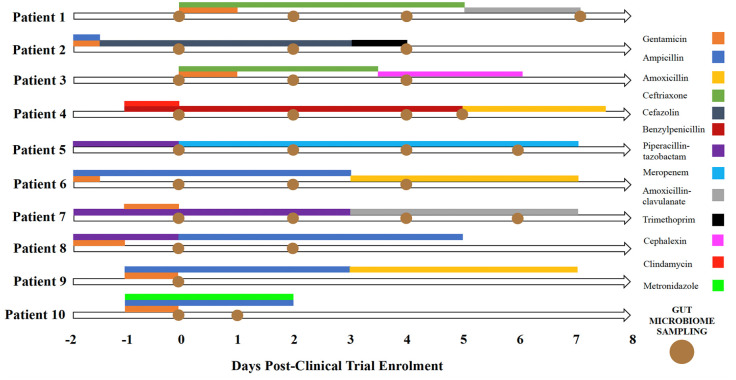
Patient timeline of gut microbiome antimicrobial exposure and faecal sampling.

Ten patients were successfully enrolled and randomised into two clinical trials (Table 1). Age ranged from 20 to 78 years old, and 6 of 10 patients were female. The bloodstream isolates encountered were *Escherichia coli*, *Streptococcus agalactiae*, *Klebsiella pneumoniae* and *Citrobacter koseri*. The trial antibiotics administered for directed therapy for bloodstream infection were ampicillin, ceftriaxone, cefazolin, benzylpenicillin, piperacillin-tazobactam and meropenem. Empiric antimicrobial therapy was heterogeneous among patients, and there was no washout period. These antibiotic regimens included ampicillin plus gentamicin, clindamycin plus benzylpenicillin, piperacillin-tazobactam, piperacillin plus gentamicin and ampicillin plus gentamicin plus metronidazole (Figure 1). One patient was pregnant (Patient 10). No patient received nutrition via an enteral feeding tube. The patient timeline of gut microbiome antimicrobial exposure and faecal sampling is outlined in Figure 1.

**Table 1.** Summary of clinical data.

Patient Number	Age	Sex	Bloodstream isolate	Source of bacteraemia	Trial	Trial antibiotic	Comorbidities	Relevant medications
Patient 1	55	Female	*E. coli*	Urinary tract infection	GAMECHANGER	Ceftriaxone	Malignancy	Chemotherapy
Patient 2	59	Female	*E. coli*	Urinary tract infection	BALANCE	Cefazolin	Diabetes mellitus Alcoholism	Pantoprazole
Patient 3	70	Female	*K. pneumoniae*	Urinary tract infection	BALANCE	Ceftriaxone	Diabetes mellitus	Empagliflozin
Patient 4	61	Female	*S. agalactiae*	Cellulitis	BALANCE	Benzylpenicillin	—	Pantoprazole
Patient 5	78	Male	*E. cloacae*	Diabetic foot ulcer	GAMECHANGER	Meropenem	Diabetes mellitus	Metformin
Patient 6	72	Female	*E. coli*	Urinary tract infection	BALANCE	Ampicillin	—	—
Patient 7	67	Male	*C. koseri*	Urinary tract infection	GAMECHANGER	Piperacillin-tazobactam	Malignancy	Rosuvastatin
Patient 8	69	Male	*E. coli*	Line-related infection	BALANCE	Ampicillin	Chronic kidney disease Diabetes mellitus	—
Patient 9	58	Male	*E. coli*	Urinary tract infection	BALANCE	Ampicillin	Diabetes mellitus	Empagliflozin Metformin
Patient 10	20	Female	*S. agalactiae*	Endometritis	BALANCE	Ampicillin	—	—

### Faecal sampling quality

Four patients produced four faecal specimens, three produced three specimens, two produced two specimens and one produced one specimen during their inpatient stay in the hospital whilst enrolled in the clinical trial. Of a total of 30 specimens, 15 were rectal swabs and 15 were stool samples. One patient had their stool specimens described as watery, whilst the rest were formed. Faecal sampling quality was determined for all 30 specimens (Table 2). Overall, DNA concentration was lower for rectal swabs compared to stool samples (16.35 vs 29.70 ng/µL). We determined that stool specimens had higher biomass than rectal swabs and, on average, yielded a much lower percentage of human (0.22% vs 24.78%) and skin flora (4.36% vs 16.37%) reads.

**Table 2.** Quality of faecal specimens among patients enrolled in a clinical trial.

Number	Sample	Description	DNA concentration (ng/µL)	Total number of reads	Human reads	Human reads (% total)	Skin flora reads*	Skin flora reads* (% total)	Unassigned reads	Analysed reads
Patient 1	Faeces	—	2.40	11,832,836	47,579	0.40	4265	0.06	5,041,588	6,743,669
Patient 1	Faeces	Watery	1.47	12,568,571	132,100	1.05	3850	0.05	5,036,626	7,399,845
Patient 1	Faeces	Mucous	0.92	10,203,057	90,508	0.89	421,934	4.88	1,564,803	8,547,746
Patient 1	Faeces	Watery	1.04	—	—	—	—	—	—	—
Patient 2	Faeces	—	23.95	10,901,394	1410	0.01	364	0	658,176	10,241,808
**Patient 2**	**Swab**	**—**	**17.71**	**11,245,386**	**3,260,199**	**28.99**	**6089**	**0.1**	**4,984,714**	**3,000,473**
Patient 2	Faeces	—	78.28	7,002,617	910	0.01	447	0.01	1,065,392	5,936,315
**Patient 3**	**Swab**	**—**	**67.16**	**9,747,874**	**7,303,746**	**74.93**	**1389**	**0.01**	**413,321**	**2,030,807**
Patient 3	Faeces	—	13.54	22,693,170	6897	0.03	4869	0.03	6,261,248	16,425,025
**Patient 3**	**Swab**	**—**	**23.65**	**18,326,408**	**15,485,673**	**84.50**	**1,482,455**	**8.3**	**472,688**	**2,368,047**
**Patient 4**	**Swab**	**Nothing**	**<Min**	**1,874,789**	**245,709**	**13.11**	**365,064**	**23.29**	**307,294**	**1,321,786**
Patient 4	Faeces	—	121.00	15,926,500	7980	0.05	3560	0.03	5,234,170	10,684,350
Patient 4	Faeces	—	54.17	14,372,247	18,651	0.13	3285	0.03	2,667,210	11,686,386
Patient 4	Faeces	—	90.59	15,543,356	4426	0.03	1940	0.01	2,484,702	13,054,228
**Patient 5**	**Swab**	**—**	**98.34**	**29,150,406**	**44,421**	**0.15**	**421,153**	**2.80**	**14,094,521**	**15,011,464**
**Patient 5**	**Swab**	—	**12.39**	**16,469,346**	**73,096**	**0.44**	**3,134,027**	**43.73**	**9,302,320**	**7,093,930**
**Patient 5**	**Swab**	**—**	**12.08**	**13,338,803**	**2,934,736**	**22.00**	**1,204,425**	**13.89**	**4,667,158**	**5,736,909**
**Patient 5**	**Swab**	—	**0.33**	**13,578,053**	**6,827,123**	**50.28**	**2,287,295**	**18.56**	**1,255,906**	**5,495,024**
**Patient 6**	**Swab**	**—**	**0.34**	**3,869,873**	**24,521**	**0.63**	**17,816**	**1.13**	**2,298,294**	**1,547,058**
Patient 6	Faeces	—	17.55	59,576,581	28,831	0.05	6530	0.02	16,496,019	43,051,731
**Patient 6**	**Swab**	**—**	**1.421**	**23,929,022**	**5,397,020**	**22.55**	**9205**	**0.05**	**4,808,883**	**13,723,119**
**Patient 7**	**Swab**	—	**3.526**	**3,998,469**	**1,646,437**	**41.2**	**1,203,200**	**34.24**	**484,575**	**1,867,457**
**Patient 7**	**Swab**	**—**	**0.6462**	**5,747,781**	**1,472,373**	**25.6**	**998,711**	**35.79**	**2,957,495**	**1,317,913**
**Patient 7**	**Swab**	—	**1.063**	**13,431,765**	**601,784**	**4.48**	**6,647,482**	**55.15**	**1,378,979**	**11,451,002**
Patient 7	Faeces	**—**	9.259	24,626,061	25,896	0.11	12,686,718	55.78	1,882,951	22,717,214
Patient 8	Faeces	—	8.363	7,976,046	13,852	0.17	2265	0.04	1,697,467	6,264,727
Patient 8	Faeces	**—**	8.284	7,759,316	17,069	0.22	1690	0.03	1,464,189	6,278,058
**Patient 9**	**Swab**	—	**1.91**	**7,534,548**	**38,058**	**0.51**	**217,567**	**3.74**	**1,719,016**	**5,777,474**
**Patient 10**	**Swab**	**—**	**4.617**	**7,389,252**	**167,742**	**2.27**	**191,717**	**4.80**	**3,397,662**	**3,823,848**
Patient 10	Faeces	—	14.62	10,109,086	3240	0.03	4284	0.09	5,121,055	4,984,791

* Skin flora contains bacterial genera *Staphylococcus*, *Propionibacterium*, *Micrococcus*, *Cutibacterium* and *Corynebacterium.*

### Community profiling and antimicrobial resistance gene abundance

**Figure 2.**
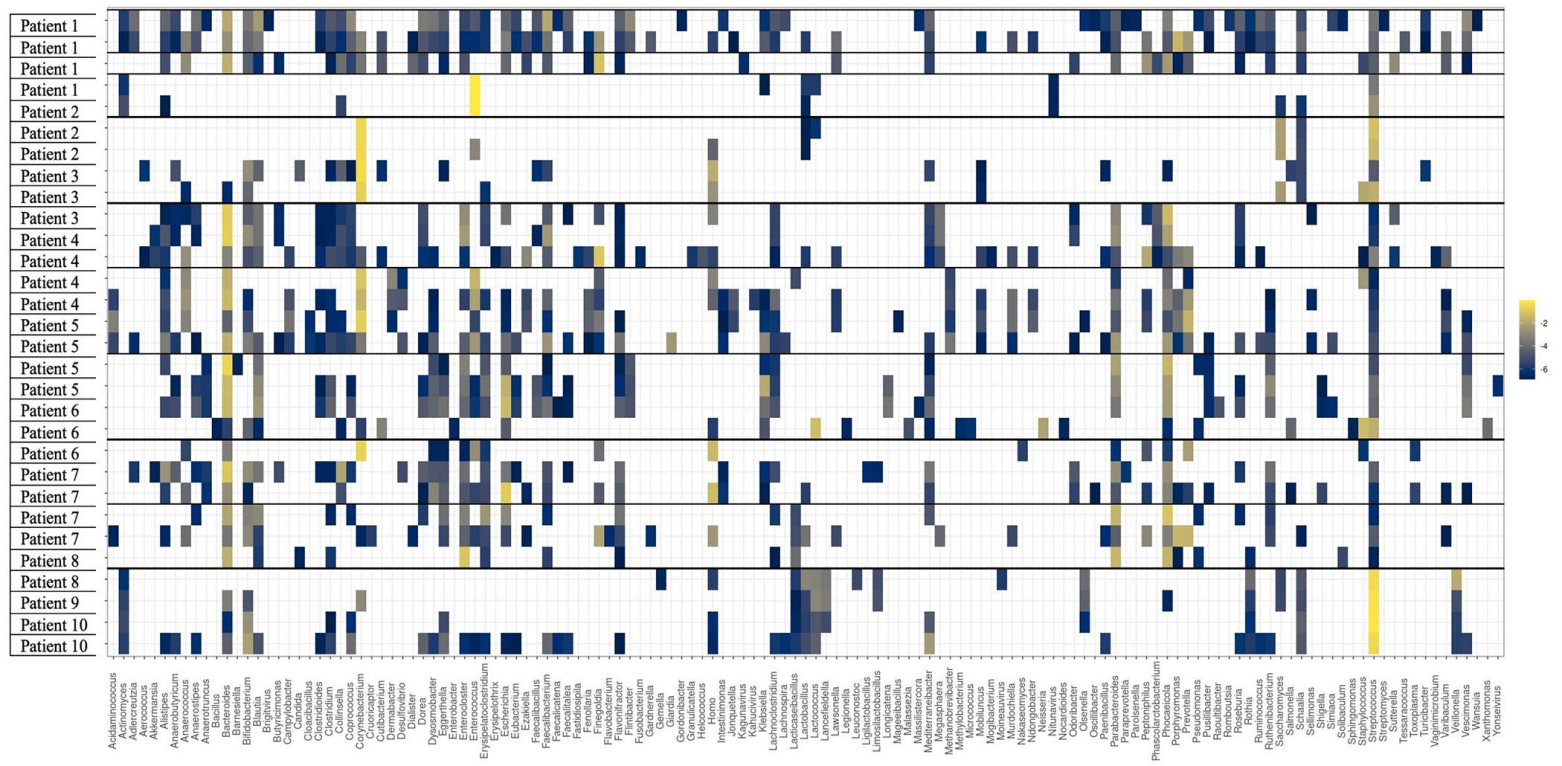
Community profiling of stool specimens collected from hospitalised patients enrolled in a clinical trial.

**Figure 3.**
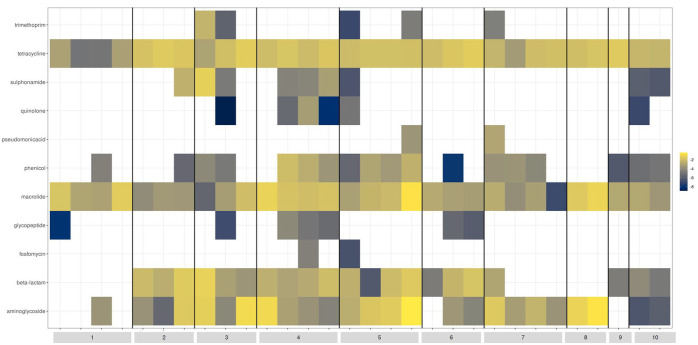
Antimicrobial resistance gene abundance in stool specimens collected from hospitalised patients enrolled in a clinical trial.

All stool specimens collected underwent community profiling and ARG abundance determination (Figures 2 and 3). Of trial participants receiving ampicillin (Patients 6, 8, 9 and 10), Patients 6, 9 and 10 demonstrated preservation of key anaerobic bacteria (*Parabacteroides* spp., *Bifidobacterium* spp., *Bacteroides* spp.). These patients also showed a marked reduction in *Prevotella* spp., *Streptococcus* spp., *Lactobacillus* spp. and *Actinomyces* spp. *Enterococcus faecium* became present or more abundant in Patients 8 and 10. *Clostridioides difficile* became more abundant in Patient 10. A reduction in the abundance of yeast (*Saccharomyces* spp.) was observed in Patient 8. An increase in abundance of *bla*_TEM—1A_ was seen in Patient 6. Of trial participants receiving ceftriaxone (Patients 1 and 3), Patient 3 demonstrated a marked reduction in Enterobacterales (including *E. coli*) in addition to the beta-lactamase gene *bla*_TEM-1A_. *Clostridioides difficile* and *E. faecalis* became present or more abundant during antimicrobial administration. Patient 1 had no beta-lactamase genes detected and exhibited a marked reduction in anaerobic species (*Bifidobacterium* spp., *Clostridium* spp., *Bacteroides* spp.) in the last specimen collection which coincided with receipt of amoxicillin-clavulanate 24 h prior. There were no ESBL genes detected in trial participants receiving ceftriaxone. Of trial participants receiving piperacillin-tazobactam (Patient 7), *E. faecium* and yeast species (*Candida* spp. and *Saccharomyces* spp.) became present or increased in abundance, coincident with a reduction in key anaerobic species (*Clostridium* spp. and *Bifidobacterium* spp.) and the abundance of *bla*_Z_ gene (Penicillinase of *Staphylococcus aureus*). Of trial participants receiving meropenem (Patient 5), there was an increased abundance of *E. faecium* and *Bacteroides* spp. with a coincident reduction in Enterobacterales and other anaerobic species (*Parabacteroides* spp., *Clostridium* spp. and *Bifidobacterium* spp.). Of note, this patient also received piperacillin-tazobactam for 3 days prior to commencement of meropenem. A marked reduction in the abundance of certain beta-lactamase genes (*bla*_SHV-100_, *bla*_OXA-392_, *bla*_CMY-18_) when transitioning from piperacillin-tazobactam to meropenem. A marked increase in abundance of the *cfxA* gene (encoding CfxA beta-lactamase) was also noted. There were no carbapenemase genes detected. Of trial participants receiving cefazolin (Patient 2), an increase in key anaerobic species (*Bifidobacterium* spp., *Clostridium* spp., *Bacteroides* spp. and *Anaerococcus* spp.) and abundance of *bla*_TEM-1A_ was observed. Of trial participants receiving benzylpenicillin (Patient 4), an increased abundance of Enterobacterales and anaerobic species (*Parabacteroides* spp., *Clostridium* spp., *Bacteroides* spp.) and of certain beta-lactamase genes (*bla*_Z_, *bla*_SHV_) and the penA gene (encoding PLP2, which is associated with decreased susceptibilities to extended-spectrum cephalosporins in gonococcal strains). A reduction in *Bifidobacterium* spp., *Lactococcus* spp. and *Streptococcus* spp. was also seen in this patient. Of patients who received at least one dose empiric gentamicin (Patients 1, 2, 3, 6, 7, 8, 9 and 10), an increase in abundance of aminoglycoside resistance genes (*aph(6)-Id, aph(3’)-Ia, aph(3’)-Ib, aac(3)-XI*) were observed in faecal specimens following exposure.

### Diversity and richness

**Figure 4.**
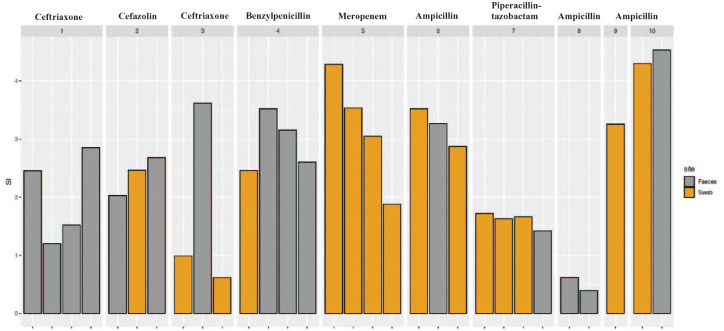
Intra- and inter-patient differences in the Shannon index among clinical trial patients receiving different antimicrobials.

The Shannon index for alpha diversity was calculated for each specimen (Figure 4). The most dramatic decline in the Shannon index (4.286 to 1.880) was seen in Patient 5 receiving meropenem. Patients exposed to antimicrobials with negligible anaerobic activity (cefazolin, ceftriaxone; Patients 1, 2 and 3) demonstrated good preservation of diversity and richness indices. Similar high Shannon indices were noted for patients exposed to ampicillin, except for Patient 8 who received 2 days of piperacillin-tazobactam prior to enrolment into the clinical trial. Despite being exposed to metronidazole, Patient 10 had the highest recorded Shannon index values (both above 4.30).

## Discussion

This nested sub-study was undertaken from within two clinical trials and sought to identify key changes *in vivo* that occur in the gut microbiome on exposure to different antimicrobials in a controlled setting. In addition, we aimed to establish an appropriate workflow and methodology for integrating microbiome analysis into clinical trials. There was a large amount of variability in DNA concentration values between specimens, which likely reflected differences in specimen volume, collection technique, staff training, as well as specimen type. Optimisation of the pre-analytical phase of microbiome analysis would require stool samples collected by (or with direct oversight from) a trained trial coordinator. Because specimens were batch tested, we were unable to accurately assess the concentration of DNA yielded in relation to time from collection to DNA extraction. Enhanced degradation of nucleic acid resulting from improper or prolonged storage has been well documented in the literature.^14,15^

Our study was able to successfully link clinically important gut microbiome changes to individual antibiotic exposure in a clinical trial setting. Relative abundance of *E. faecium*, which is an ESKAPE pathogen (acronym of: *E. faecium, S. aureus, K. pneumoniae, A. baumannii, P. aeruginosa* and *Enterobacter* species) responsible for many healthcare-associated infections^16^ was observed to increase in individual patients that received ampicillin, meropenem and piperacillin-tazobactam.^16^ There were no *van* genes detected. Although thought to cause comparatively less collateral damage, ampicillin may target specific anaerobes, Enterobacterales and *E. faecalis*, culminating in an environment that enables the expansion of *E. faecium*.^17^ This finding has important antimicrobial stewardship and infection control implications and thus requires further investigation. *C. difficile* was detected in two patients, one who received a combination of ampicillin, gentamicin and metronidazole and the other with ceftriaxone. Although metronidazole has been described as a protective factor against *C. difficile* infection, colonisation resistance from ampicillin and gentamicin, favouring expansion of *C. difficile*, may have overcome this effect.^18^ Pre-clinical models have been able to shed light on the precise gut microbiome perturbations that lead to *C. difficile* infection.^19^ *Porphyromonadaceae*, *Lachnospiraceae*, *Lactobacillus* and *Alistipes* inhibit intestinal colonisation by *C. difficile*, whereas the genera *Escherichia* and *Streptococcus* have the opposite effect.^19^ *C. difficile* was not detected from stool samples in patients receiving piperacillin-tazobactam or meropenem, although the analytical sensitivity of targeted molecular assays (e.g., singleplex real-time PCR) is likely to be greater than for metagenomic sequencing.^20^ The differences between *C. difficile* nucleic acid detection (and quantitation) from metagenomic sequencing versus conventional testing (e.g., stool EIA and toxin PCR) with respect to clinical disease and infection control implications require investigation.

The differential depletion of anaerobic genera from the gut microbiome on exposure to antimicrobials with anti-anaerobic activity was notable. *Bifidobacterium* species are important to gut microbial diversity and were relatively preserved in patients exposed to ampicillin. Despite predictably depleting *Clostridium* and *Bifidobacterium* species, exposure to meropenem resulted in an increased abundance of *Bacteroides* species. Ambler class A beta-lactamase gene *cfxA* is associated with cephamycin resistance and is found on *Bacteroides* and *Parabacteroides* species.^21^ The *cfiA* gene in *B. fragilis* is a metallo-beta-lactamase that is responsible for carbapenem resistance and has been documented.^22^ There were no *cfiA* genes identified in our study.

With regards to beta-lactam antimicrobial resistance determinants, *bla*_TEM-1A_ abundance increased in the setting of ampicillin exposure. Ampicillin likely selected TEM-1A-producing Enterobacterales in the gut microbiota.^23^ This has direct and immediate clinical consequences in that further bloodstream or healthcare-associated infection would likely be due to a TEM-1A producer and would require therapy with a further detrimental effect on the gut microbiome (e.g., amoxicillin-clavulanate, ceftriaxone). This was made evident with Patient 3 who received ceftriaxone and demonstrated a rapid reduction in abundance of *bla*_TEM-1A_ followed by a rise in *C. difficile* and *E. faecalis*. Patient 5 received piperacillin-tazobactam in the days prior to clinical trial enrolment, followed by meropenem. The first faecal specimen at the point of antimicrobial transition demonstrated an abundance of beta-lactamase genes (*bla*_SHV-100_, *bla*_OXA-392_ and *bla*_CMY-18_), which were eradicated with subsequent meropenem exposure. Concerns over the clinical efficacy and *in vitro* activity of piperacillin-tazobactam against OXA-1 and CMY-2-producing Enterobacterales have been raised.^24,25^ Also, exposure of the gut microbiome to a certain class of antibiotics may result in resistance development to a separate class of antibiotics (i.e., cross-resistance). This has been demonstrated in a pre-clinical model.^26^

Yeast species (*Candida* spp. and *Saccharomyces* spp.) became most prevalent in the gut microbiome in the patient with piperacillin-tazobactam exposure. Although many broad-spectrum antibiotics have been associated with gastrointestinal tract colonisation with *Candida* species, piperacillin-tazobactam may be associated with a much higher comparative risk.^27^ Monitoring the risk of invasive candidiasis with piperacillin-tazobactam exposure in a clinical trial setting is important.

Integration of gut microbiome analysis into contemporary antibiotic clinical trials has been proposed to better aid treatment decisions and guide the placement of new antimicrobials in the clinical space in the post-marketing phase of development.^8^ A microbiome nested sub-study was performed in the PIRATE trial, which assessed the non-inferiority of shorter antibiotic courses (7 vs 14 days) for Gram-negative bacteraemia.^28^ Enrolled participants were followed up to 30 days, and there was no difference in ARG abundance (median counts per million) or microbial species diversity (Shannon species diversity) between the two groups. In healthy volunteers, gut microbiome diversity and richness have been reported in a study to return to pre-antibiotic treatment levels after approximately 2 months.^4^ Long-acting antibiotics (e.g., azithromycin) and pre-treatment microbiota resembling sick and hospitalised patients lead to more persistent antibiotic-related microbiome changes. Other studies have echoed the importance of pre-treatment microbiota composition.^29^ Moreover, 20% of patients in our study were taking a proton pump inhibitor, which has been independently associated with lower abundance in gut commensals and lower microbial diversity.^30^ This likely confounded findings in these patients.

There were many limitations to this study. First, the evaluable population was heterogeneous and was derived from two separate clinical trials that sought to answer two different clinical questions. Moreover, there were many trial antibiotics administered (six in total), with many having pre-trial exposure to different antimicrobials altogether. None of the patients received only a single antimicrobial, and the flow-on effect from a previous antimicrobial agent could have contributed to findings. In addition, no patients had a faecal specimen taken prior to antibiotic treatment, thus limiting our interpretations of the data. Although this represents a “real-world” setting, it creates difficulty in defining true antibiotic-associated effects on the gut microbiome. Second, faecal sampling number and duration were suboptimal, and specimens were batch tested. Gut microbiome adaptations to antimicrobials are known to evolve over time, which requires analysis over weeks to months to get an accurate picture. From a feasibility standpoint, it is much easier to collect faecal specimens from patients admitted to the hospital. This needs to be weighed up with every study where collecting quality stool samples from the community may cause additional issues with the study (i.e., participant response rates, storage and transport of specimens and additional costs). A proposed solution to this has been the development of commercial self-collection kits with in-built DNA/RNA stabilisers, which prevent nucleic acid degradation and enable prolonged storage and transport times. Third, half of all specimens collected were from rectal swabs, which proved to produce more human and skin flora sequences using the techniques and swab type in this study. Although easier to collect and perhaps more acceptable to patients, this needs to be weighed against study quality. Fourth, this study only recruited 10 patients and therefore did not have enough power to detect gut microbiome effects that may be evident with certain antimicrobials. By having large groups of individuals being exposed to the same antimicrobial in a clinical trial setting, this would allow for statistical analysis, which could result in a more refined understanding.

## Conclusion

Understanding the determinants of antimicrobial resistance development at an individual level is of great clinical importance. Stool samples provide higher biomass samples for sequencing and should be considered the preferred specimen type obtained for these studies. A number of challenges exist with regards to integrating gut microbiome analysis into clinical trials (e.g., obtaining an antibiotic-free stool specimen at baseline). Narrow-spectrum antibiotics may have a greater, and more clinically important, effect on our gut microbiota than once thought. The presence of key anaerobes such as *Clostridium* and *Bifidobacterium* species may play an important role in colonisation resistance and may be suitable outcome measures for future clinical trials. Further refinement of clinical trial infrastructure, methodology and workflow is required before existing challenges can be overcome.

## Declarations

**Ethics approval and consent to participate:** This study was approved by the Royal Brisbane & Women’s Hospital Human Research Ethics Committee (LNR/2020/QRBW/65029). *Written informed consent for inclusion in our study was obtained from participants*.

**Consent for publication:** We have obtained written informed consent from the participants for the publication of this study as basic identifiers (age and sex) present in Table 1.

**Availability of data and materials:** The minimal dataset is available within the paper. Additional data is available upon reasonable requests from the corresponding author (Dr Adam Stewart) at adam.stewart@uq.edu.au.

## References

[1] Antimicrobial Resistance Collaborators. Global burden of bacterial antimicrobial resistance in 2019: a systematic analysis. Lancet 2022; 399(10325): 629–655. 35065702 10.1016/S0140-6736(21)02724-0 PMC8841637

[2] Fishbein SRS Mahmud B Dantas G. Antibiotic perturbations to the gut microbiome. Nat Rev Microbiol 2023; 21: 772–788. 37491458 10.1038/s41579-023-00933-y PMC12087466

[3] Maier L Goemans CV Wirbel J , et al. Unravelling the collateral damage of antibiotics on gut bacteria. Nature 2021; 599(7883): 120–124. 34646011 10.1038/s41586-021-03986-2 PMC7612847

[4] Anthony WE Wang B Sukhum KV , et al. Acute and persistent effects of commonly used antibiotics on the gut microbiome and resistome in healthy adults. Cell Rep 2022; 39(2): 110649. 35417701 10.1016/j.celrep.2022.110649 PMC9066705

[5] Patangia DV Anthony Ryan C Dempsey E , et al. Impact of antibiotics on the human microbiome and consequences for host health. Microbiologyopen 2022; 11(1): e1260. 10.1002/mbo3.1260 PMC8756738 35212478

[6] Sorbara MT Pamer EG. Interbacterial mechanisms of colonization resistance and the strategies pathogens use to overcome them. Mucosal Immunol 2019; 12(1): 1–9. 29988120 10.1038/s41385-018-0053-0 PMC6312114

[7] Knight R Vrbanac A Taylor BC , et al. Best practices for analysing microbiomes. Nat Rev Microbiol 2018; 16(7): 410–422. 29795328 10.1038/s41579-018-0029-9

[8] Stewart AG Satlin MJ Schlebusch S , et al. Completing the picture-capturing the resistome in antibiotic clinical trials. Clin Infect Dis 2021; 72(12): e1122–e1129. 10.1093/cid/ciaa1877 33354717

[9] Schlebusch S Graham RMA Jennison AV , et al. Standard rectal swabs as a surrogate sample for gut microbiome monitoring in intensive care. BMC Microbiol 2022; 22(1): 99. 35413802 10.1186/s12866-022-02487-0 PMC9004175

[10] Langmead B Salzberg SL. Fast gapped-read alignment with Bowtie 2. Nat Methods 2012; 9(4): 357–359. 22388286 10.1038/nmeth.1923 PMC3322381

[11] Petersen TN Lukjancenko O Frølund Thomsen MC , et al. MGmapper: reference based mapping and taxonomy annotation of metagenomics sequence reads. PLoS One 2017; 12(5): e0176469. 10.1371/journal.pone.0176469 PMC5415185 28467460

[12] Florensa AF Sommer Kaas R Thomas Lanken Conradsen Clausen P , et al. ResFinder - an open online resource for identification of antimicrobial resistance genes in next-generation sequencing data and prediction of phenotypes from genotypes. Microb Genom 2022; 8(1): 000748. 35072601 10.1099/mgen.0.000748 PMC8914360

[13] Wood DE Salzberg SL. Kraken: ultrafast metagenomic sequence classification using exact alignments. Genome Biol 2014; 15(3): R46. 10.1186/gb-2014-15-3-r46 PMC4053813 24580807

[14] Thomas V Clark J Doré J. Fecal microbiota analysis: an overview of sample collection methods and sequencing strategies. Future Microbiol 2015; 10(9): 1485–1504. 26347019 10.2217/fmb.15.87

[15] Fouhy F Deane J Rea MC , et al. The effects of freezing on faecal microbiota as determined using MiSeq sequencing and culture-based investigations. PLoS One 2015; 10(3): e0119355. 10.1371/journal.pone.0119355 PMC4352061 25748176

[16] De Oliveira DMP Forde BM Kidd TJ , et al. Antimicrobial resistance in ESKAPE pathogens. Clin Microbiol Rev 2020; 33(3): e00181–19. 10.1128/CMR.00181-19 PMC7227449 32404435

[17] Dubin K Pamer EG. Enterococci and their interactions with the intestinal microbiome. Microbiol Spectr 2014; 5(6): bad-0014-2016. 10.1128/microbiolspec.bad-0014-2016 PMC5691600 29125098

[18] Cui Y Dong D Zhang L , et al. Risk factors for Clostridioides difficile infection and colonization among patients admitted to an intensive care unit in Shanghai, China. BMC Infect Dis 2019; 19(1): 961. 31711425 10.1186/s12879-019-4603-1 PMC6849324

[19] Schubert AM Sinani H Schloss PD. Antibiotic-induced alterations of the murine gut microbiota and subsequent effects on colonization resistance against clostridium difficile. mBio 2015; 6(4): e00974. 10.1128/mBio.00974-15 PMC4502226 26173701

[20] Ferreira C Otani S Møller Aarestrup F , et al. Quantitative PCR versus metagenomics for monitoring antibiotic resistance genes: balancing high sensitivity and broad coverage. FEMS Microbes 2023; 4: xtad008. 10.1093/femsmc/xtad008 PMC10117749 37333442

[21] Rong SMM Rodloff AC Stingu CS. Diversity of antimicrobial resistance genes in bacteroides and parabacteroides strains isolated in Germany. J Glob Antimicrob Resist 2021; 24: 328–334. 33508481 10.1016/j.jgar.2021.01.007

[22] Kierzkowska M Majewska A Karłowicz K , et al. Phenotypic and genotypic identification of carbapenem resistance in Bacteroides fragilis clinical strains. Med Microbiol Immunol 2023; 212(3): 231–240. 37178261 10.1007/s00430-023-00765-w PMC10293361

[23] Livermore DM Moosdeen F Lindridge MA , et al. Behaviour of TEM-1 beta-lactamase as a resistance mechanism to ampicillin, mezlocillin and azlocillin in Escherichia coli. J Antimicrob Chemother 1986; 17(2): 139–146. 3516962 10.1093/jac/17.2.139

[24] Stewart AG Paterson DL Young B , et al. Meropenem versus piperacillin-tazobactam for definitive treatment of bloodstream infections caused by ampC β-lactamase-producing Enterobacter spp, Citrobacter freundii, Morganella morganii, Providencia spp, or Serratia marcescens: a pilot multicenter randomized controlled trial (MERINO-2). Open Forum Infect Dis 2021; 8(8): ofab387. 10.1093/ofid/ofab387 PMC8361238 34395716

[25] Henderson A . Association with 30-day mortality and MIC in patients treated with piperacillin/tazobactam for Escherichia coli and Klebsiella pneumoniae bloodstream infections that are non-susceptible to ceftriaxone from patients enrolled in the MERINO trial. In: 29th European congress of clinical microbiology & infectious diseases, Amsterdam, Netherlands, 2019.

[26] Xu L Surathu A Raplee I , et al. The effect of antibiotics on the gut microbiome: a metagenomics analysis of microbial shift and gut antibiotic resistance in antibiotic treated mice. BMC Genomics 2020; 21(1): 263. 32228448 10.1186/s12864-020-6665-2 PMC7106814

[27] Lin MY Carmeli Y Zumsteg J , et al. Prior antimicrobial therapy and risk for hospital-acquired Candida glabrata and Candida krusei fungemia: a case-case-control study. Antimicrob Agents Chemother 2005; 49(11): 4555–4560. 16251295 10.1128/AAC.49.11.4555-4560.2005 PMC1280123

[28] Leo S Lazarevic V von Dach E , et al. Effects of antibiotic duration on the intestinal microbiota and resistome: the PIRATE RESISTANCE project, a cohort study nested within a randomized trial. EBioMedicine 2021; 71: 103566. 34492446 10.1016/j.ebiom.2021.103566 PMC8426194

[29] Rashidi A Ebadi M Rehman TU , et al. Gut microbiota response to antibiotics is personalized and depends on baseline microbiota. Microbiome 2021; 9(1): 211. 34702350 10.1186/s40168-021-01170-2 PMC8549152

[30] Jackson MA Goodrich JK Maxan M-E , et al. Proton pump inhibitors alter the composition of the gut microbiota. Gut 2016; 65(5): 749–756. 26719299 10.1136/gutjnl-2015-310861 PMC4853574

